# Orally active prostacyclin analogue beraprost sodium in patients with chronic kidney disease: a randomized, double-blind, placebo-controlled, phase II dose finding trial

**DOI:** 10.1186/s12882-015-0130-5

**Published:** 2015-10-16

**Authors:** Akio Koyama, Toshiro Fujita, Fumitake Gejyo, Hideki Origasa, Masanao Isono, Hajimu Kurumatani, Kiyonobu Okada, Hiroyuki Kanoh, Takashi Kiriyama, Shunsuke Yamada

**Affiliations:** University of Tsukuba, 1-1-1 Tennodai, Tsukuba, Ibaraki 305-8577 Japan; Division of Clinical Epigenetics, Research Center for Advanced Science and Technology, The University of Tokyo, 4-6-1 Komaba, Meguro-ku, Tokyo 153-8904 Japan; Niigata University, 1-757, Asahimachi-dori, Chuo-ku, Niigata 951-8510 Japan; Division of Biostatistics and Clinical Epidemiology, Graduate School of Medicine and Pharmaceutical Sciences, The University of Toyama, 2630 Sugitani, Toyama City, 930-0194 Japan; Toray Industries, Inc., 1-1, Nihonbashi-Muromachi 2-chome, Chuo-ku, Tokyo 103-8666 Japan; Astellas Pharma Inc., 2-5-1 Nihonbashi-honcho, Chuo-ku, Tokyo 103-8411 Japan

**Keywords:** Beraprost sodium, Prostacyclin analogue, CKD, TRK-100STP, Phase II trial

## Abstract

**Background:**

Evidence increasingly points to the importance of chronic hypoxia in the tubulointerstitium as a final common pathway to end-stage renal disease (ESRD). Beraprost sodium (BPS) is an orally active prostacyclin (PGI_2_) analogue demonstrating prevention of the progression of chronic kidney disease (CKD) in various animal models by maintaining renal blood flow and attenuating renal ischemic condition.

**Methods:**

This multicenter, randomized, double-blind, placebo-controlled, phase II trial was designed to determine the recommended dose of the sustained-release form of BPS (TRK-100STP 120 μg/day or 240 μg/day) in Japanese patients with CKD. TRK-100STP was administered to a total of 112 patients. The primary efficacy endpoint was the difference in the slope of the regression line of reciprocal of serum creatinine (1/SCr) over time, obtained by the least-squares method.

**Results:**

Regarding the primary endpoint, statistical superiority of TRK-100STP 240 μg over placebo was not confirmed and so a recommended dose was not determined. Compared to placebo, however, the slope of regression line of 1/SCr, elevation of SCr and serum cystatin C during the treatment period revealed greater improvement at 120 μg, at both doses, and at 240 μg, respectively. In terms of safety, both TRK-100STP treatment groups were well tolerated.

**Conclusions:**

Although the study failed to meet the primary endpoint, results indicate that TRK-100STP may potentially prevent the decline in renal function of CKD patients independent of blood pressure or urinary protein levels.

**Trial registration:**

NCT02480751. June 21, 2015.

**Electronic supplementary material:**

The online version of this article (doi:10.1186/s12882-015-0130-5) contains supplementary material, which is available to authorized users.

## Background

An increasing number of patients with end-stage renal disease (ESRD) require dialysis or transplantation. Although diabetic nephropathy is a major reason for eventual ESRD, primary glomerular diseases and nephrosclerosis still comprise significant proportions of chronic kidney disease (CKD) patients, especially in Asian countries [1–4].

Angiotensin-converting enzyme inhibitors (ACEIs) and angiotensin-II receptor blockers (ARBs) are often administered to both diabetic and non-diabetic nephropathy patients, and are established as recommended treatment agents for non-diabetic nephropathy patients with albumin excretion [5]. However, it is evident that these agents are insufficient for the prevention of progressive renal disease. In addition, the use of combination therapy of renin-angiotensin system (RAS) inhibitors such as ACEI and ARB has demonstrated in some recent clinical trials to be not effective [6–9]. These findings suggest an urgent clinical need for a new treatment option able to significantly delay the progression of CKD.

Prostacyclin (PGI_2_) is primarily synthesized in endothelial cells and one of the important functions is to protect kidneys from ischemic damage in pathophysiological conditions [10], as evidenced, for example, by the development of renal impairment in prostacyclin synthase knockout mice [11]. Attempts have been made to use PGI_2_ or its analogues in patients with kidney disease; the PGI_2_ analogue iloprost was successfully used for patients with contrast media-induced nephropathy [12].

Beraprost sodium (BPS) is an orally active PGI_2_ analogue [13] and TRK-100STP is its sustained-release form [14]; both were generated and developed by Toray Industries, Inc. In Asian countries, the immediate-release form of BPS has been widely used in the treatment of patients with chronic arterial occlusion and pulmonary arterial hypertension [15].

Recent evidence increasingly points to the importance of chronic hypoxia especially in the tubulointerstitium as a final common pathway to the progression of CKD [16–20]. BPS prevents the progression of CKD in various animal models [21–24] by maintaining renal microvasculature and blood flow [25, 26]. In addition, it is reported that BPS is effective in Acute Kidney Injury such as contrast nephropathy and cisplatin nephropathy [27, 28]. These effects of BPS are thought to be based on multiple mechanisms of actions: direct protective effect on vascular endothelial cells [29]; inhibition of the production of inflammatory cytokines from monocyte/macrophages [23]; vasodilative [30] and antiplatelet effects [31]. The effects of BPS have also been assessed in two studies in patients with glomerulonephritis [32, 33]. Although these studies were open-label and did not have control group, the results of one study suggested that BPS mitigates the progression rate of renal dysfunction [32, 33] by increasing renal blood flow without glomerular hyperfiltration.

The efficacy of BPS on diabetic nephropathy has also been reported in several clinical [34] and non-clinical studies [35–37]; however, several methodological limitation are evident with regards to the involvement of both diabetic and non-diabetic CKD patients in a single protocol. Given the evident unmet need for non-diabetic nephropathy treatment in Asia and considering that much of the non-clinical data on BPS has been generated in non-diabetic CKD, the priority and focus of the present study was on non-diabetic CKD, given that renal disorders are not modified by diabetes. This is the first randomized, placebo-controlled, double-blind, comparative trial to investigate the recommended dose at which TRK-100STP suppresses the progression of CKD in patients with primary glomerular disease or nephrosclerosis.

## Methods

This multicenter, randomized, double-blind placebo-controlled, phase II trial was designed to determine the recommended dose of TRK-100STP (i.e., either 120 μg/day or 240 μg/day) for suppressing progression in Japanese patients with primary glomerular disease or nephrosclerosis. The study was approved by each participating center’s local Research Ethics Committee. Only patients with written informed consent were included (Please refer to the list of participating centers at the end of the paper).

### Study design

**Fig. 1:**
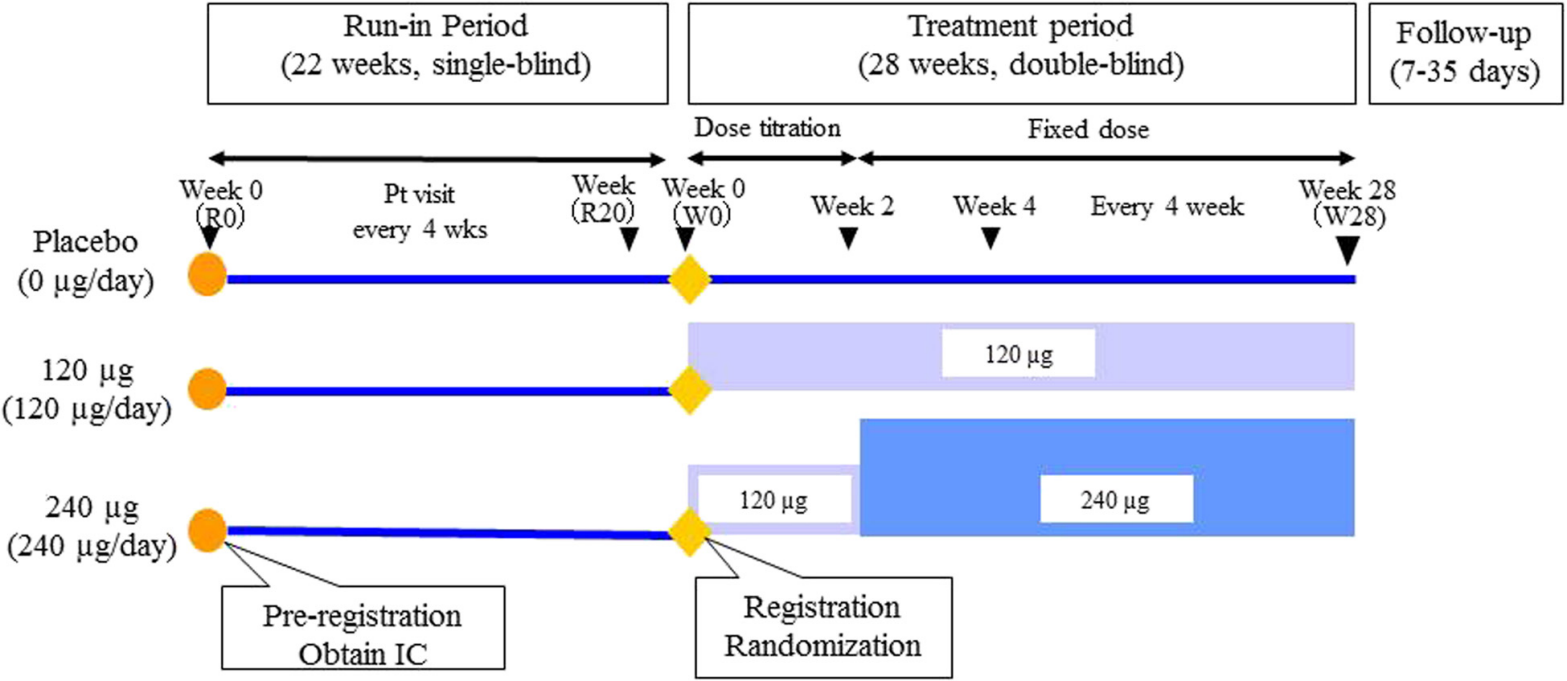
Summary of study design

**Fig. 2:**
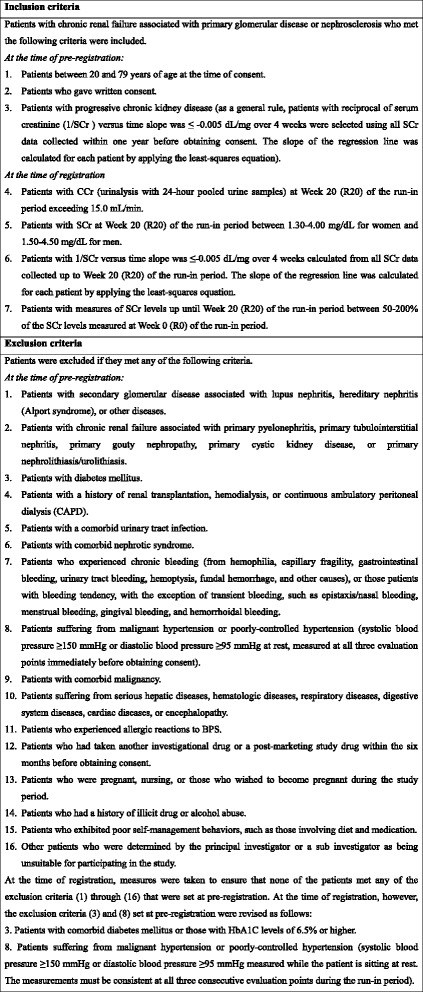
Inclusion and exclusion cliteria

A summary of the study method is presented in Fig. 1. After the run-in period, when placebo tablets were orally administered twice daily for 22 weeks in a single-blinded manner, patients who met all of the inclusion criteria and none of the exclusion criteria (detailed in Fig. 2) were randomized into one of the three treatment groups (TRK-100STP 120 μg, 240 μg or placebo group) using computer random number generator to select random permuted blocks. Central randomization was performed by external statistician for the 28-week treatment period in a double-blinded manner. Enrollment to the trial was restricted to patients whose renal function had progressively declined during the 22-week run-in period (R0 ~ R20) to obtain adequate treatment response data during the 28-week treatment period (W0 ~ W28).

Reciprocal of serum creatinine (1/SCr) versus time slope shows the speed of renal function decline so that this parameter is used to estimate how long it will take to start dialysis and to evaluate therapeutic effects [38]. This parameter is used for example to show the status of nephropathy in the RENAAL trial [39].

Therefore, in order to limit the participants to those with progressive diseases, only patients whose reciprocal of serum creatinine (1/SCr) versus time slope was ≤ −0.005 dL/mg over 4 weeks during the run-in period were randomized. This value was based on the patients’ background in a trial of orally administered spherical carbon adsorbent AST-120 [40]. The primary endpoint was also 1/SCr versus time slope and the differences between the treatment- and the observation-period values were evaluated.

The study drug in the treatment period was orally administered twice daily, after meals, for 28 weeks in a double-blinded manner. The first 2 weeks of the treatment period were designated as the dose-titration period with respect to safety analyses. The initial dose for the TRK-100STP groups was 120 μg/day. For the 240 μg group, the dose was increased to 240 μg/day after the 2-week dose-titration period. One follow-up assessment was performed between 7 to 35 days after completion or discontinuation of study drug during the treatment period.

The two doses of TRK-100STP (120 μg/day and 240 μg/day) were selected for this trial on the basis that a 30 μg single dose was considered unable to achieve the effective plasma concentration, whilst a 180 μg single dose previously resulted in a higher incidence of headache and other adverse drug reactions (ADRs) in a completed phase I clinical study of TRK-100STP. Based on these observations, TRK-100STP at 60 to 120 μg b.i.d. (i.e., 120 to 240 μg/day) were selected for investigation in the present trial.

### Study endpoints

The primary efficacy endpoint was the difference between the run-in and treatment periods in the slope of the regression line of 1/SCr versus time, which was calculated by the least-squares method. All SCr data collected in the run-in period (R0 ~ R20) and the treatment period (W4 ~ W28) were used for the plot in order to exclude the possible effect of the titration period (W0 ~ W2).

Secondary endpoints were as follows: 1) The difference between the run-in period (R0 ~ R20) and treatment period (W0 ~ W28) in 1/SCr versus time slope; 2) changes in creatinine clearance (CCr, Cockcroft-Gault equation); 3) changes in CCr (urinalysis with 24-hour pooled urine); 4) changes in urinary protein excretion; 5) ratio of SCr; and 6) changes in serum cystatin C. Safety endpoints were as follows: 1) adverse events (AEs); 2) clinical laboratory tests; 3) vital signs and body weight; and 4) 12-lead electrocardiogram (ECG).

### Prohibited and restricted concomitant medications

#### Prohibited concomitant medications

Use of the following medications were prohibited during the study period: spherical carbon adsorbent; prostaglandin analogues, other than eye drops and those in ointment form; antiplatelet agents, with the exception of aspirin products; anticoagulant and thrombolytic agents, except for temporary use such as for examinations; fluorinated pyrimidine antifungal agents; and iodinated radiocontrast agents.

#### Restricted concomitant medications

Dosage of ACEIs and ARBs were stipulated to be fixed during the study period. However, in necessary situations, such as increased serum potassium levels that did not improve with alternative options, a reduction in ACEI or ARB doses was allowed. Taking nonsteroidal anti-inflammatory drugs (NSAIDs) continuously for more than one week was prohibited.

### Sample size estimation

The sample size estimation was based on a previous study, a Phase III clinical trial of AST-120 (indicated for the treatment of chronic renal failure (CRF) in Japan), conducted in a total of 237 patients (119 patients treated with the active drug and 118 received placebo) for 24 weeks [40]. The study showed that the difference in the slope of regression line of 1/SCr versus time of AST-120 and the placebo groups before and after treatment with the test drug was 0.00352 (dL/mg over 4 weeks). Based on this value, we hypothesized that the TRK-100STP 120 μg group would show efficacy similar to the AST-120 group, and that the TRK-100STP 240 μg group would show 1.5 times better efficacy than the AST-120 group. According to these predictions, we set contrast coefficients [−1, 0, 1] for the placebo, the TRK-100STP 120 μg, and the 240 μg groups. The number of patients required per group was estimated to be 71 (with a two-sided 5 % significance level and 80 % statistical power). On this basis, the target number of patients needed to recruit was set at 430 patients, taking into account the possibility that the patient withdrawal rate in the run-in period might be high because of the long run-in period.

### Statistical analysis

(a) Primary endpoint

Using all SCr data collected during the run-in period (R0 ~ R20) and treatment period (W4 ~ W28), reciprocal SCr values plotted against time were analyzed. The slopes of the regression line were calculated by applying the least-squares equation. The difference in the slopes between the run-in period (R0 ~ R20) and treatment period (W4 ~ W28) was evaluated as the primary endpoint. In order to determine the recommended dose, the primary endpoint was analyzed by the following procedures:

- *Step 1:* Analysis of covariance (ANCOVA) with the slopes calculated from the results obtained during the run-in period as a covariate after assigning contrast coefficients [−1, 0, 1].
- *Step 2:* ANCOVA with the slopes obtained from the results collected during the run-in period as a covariate after assigning contrast coefficient [−1, 1, 0], only when a statistical significance was detected in Step 1.

(b) Secondary endpoints

Analytical methods for secondary endpoints are described in each result.

(c) Post hoc analysis

*Ratio of SCr (the final evaluation point/W0)*

In analyzing the ratio of SCr for the secondary endpoint, the SCr values measured at R20 were chosen as baseline as other parameters such as CCr, urinary protein and cystatin C were measured only at R20. Regarding SCr, however, as the value at W0 measured just before the treatment period was available, the ratio of SCr (the final evaluation point/W0) was assessed by ANCOVA with the SCr (R20) as covariate.

*Change in serum cystatin C in the treatment period (final evaluation point - R20)*

As the ratio of SCr was analyzed only for the treatment period, changes in cystatin C were re-evaluated based only on data from the treatment period.

Changes in cystatin C were calculated using values taken at R20 as the baseline (the final evaluation point – R20). ANCOVA was performed with the SCr (R20) as the covariate. As the values at W0 were not available, the values at R20, the examination point of the study closest to W0, were used as baseline.

*Ratio of eGFR (the final evaluation point/W0)*

Ratio of eGFR (the final evaluation point/W0) was assessed by ANCOVA with the SCr (R20) as covariate. In order to calculate the eGFR for Japanese patients, the following equation was used;

GFR(male) = 194*Scr^-1.094^*age^-0.287^, and GFR(female) = GFR(male)*0.739 [41].

### Study measurements

The parameters that were measured and analyzed are listed in Table 1.

**Table 1:** Study measurements

Examination by investigator	Objective symptoms (patient interview)
Physical examination	Body weight, blood pressure, pulse rate, 12-lead ECG
Hematological examination	WBC, RBC, Hb, Ht, PLT,
	Differential count of leukocytes (basophil, eosinophil, neutrophil, lymphocyte, monocyte)
Bleeding and coagulation test	PT, APTT
Blood biochemistry	TP, Alb, T-BIL, AST(GOT), ALT(GPT), ALP, LDH, γ-GTP,
	TCh, TG, UA, BUN, serum creatinine (SCr), Na, K, Cl, Ca, P, HbA1c, cystatin C,
	High-sensitive CRP
Uremia toxins test in plasma	Guanidino succinate
Urinalysis (occasional urine)	pH, qualitative protein, glucose, and urobilinogen, occult blood reaction urinary sediment (RBC, WBC, casts), β2 microglobulin (adjusted by creatinine), pregnancy test (hCG)
Urinalysis (24-hour pooled urine)	Urinary protein excretion, CCr, urea nitrogen, electrolytes (Na, Cl), urine output, creatinine

### Trial registration

NCT02480751. June 21, 2015.

## Results

### Patient disposition

The trial began on October 24, 2005 (the date the first consent was obtained) and was completed on May 20, 2008 (the last follow-up date of the final patient).

**Fig. 3:**
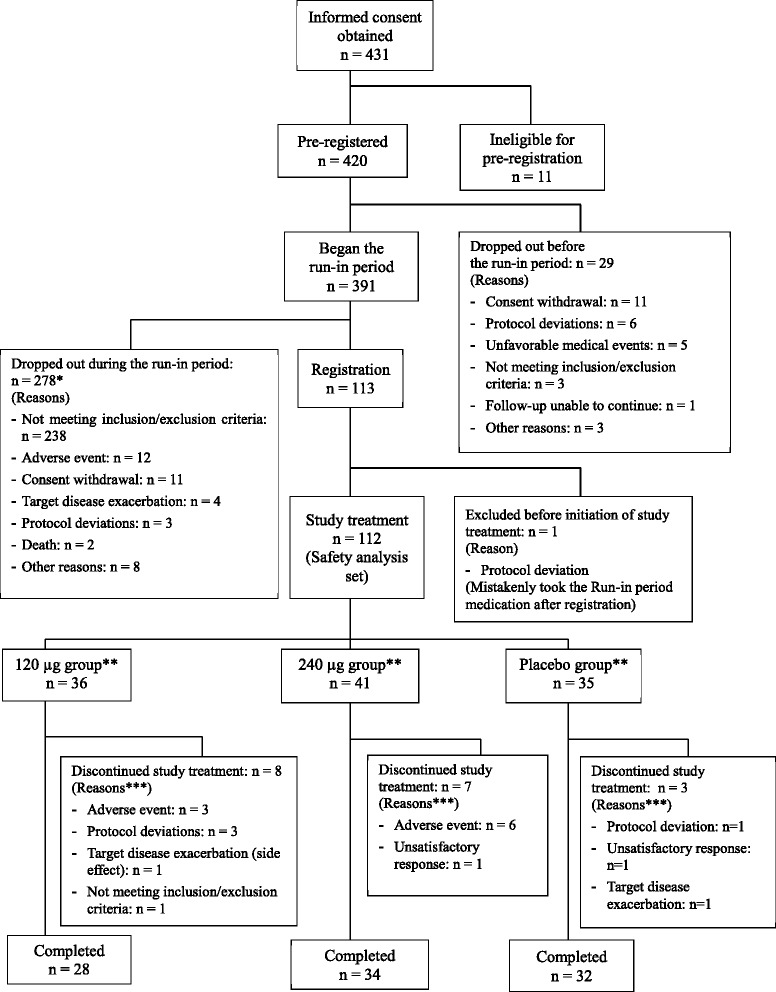
Patient dispositiona. aCONSORT 2010 flow diagram was provided as Additional file 1. * The number of the patients who were excluded during the Run-in period (n = 279) was calculated by adding the number of the patients who dropped out during the Run-in period (n = 278) and the patient who was excluded before the initiation of the study treatment (n = 1). ** Ten patients whose SCr values measured less than three points after Week 4 of the Treatment period were excluded from 112 patients randomized. As a results, 102 patients (32 patients in the 120 μg group, 36 patients in the 240 μg group and 34 patients in the placebo group) were included in the full analysis set (FAS). *** If there were multiple reasons for discontinuation, the main reason was used for calculation

A summary of the patient disposition is described in Fig. 3. Written informed consents were obtained from a total of 431 patients, 11 of whom did not meet the criteria on pre-registration and a total of 420 patients were pre-registered. Of these 420 patients, 29 dropped out before the start of the run-in period and a further 278 patients were withdrawn during the run-in period or at the registration. A total of 113 patients were therefore registered, of which one patient, who mistakenly took the run-in period medication and did not take the study drug for the treatment period after the registration, was excluded from the study. As a result, the study drugs were administered to a final total of 112 patients (35 patients in the placebo group, 36 patients in the 120 μg group and 41 patients in the 240 μg group) in a double-blind manner. Most of 238 patients who did not meet the inclusion/exclusion criteria before registration were excluded because their renal failure progressed at a slower rate during the run-in period, represented by a 1/SCr versus time slope of ≤ −0.005 dL/mg over 4 weeks.

### Analysis sets

Ten patients whose SCr values measured less than three points after Week 4 of the treatment period were excluded from 112 patients randomized. As a result, 102 patients (34 patients in the placebo group, 32 patients in the 120 μg group and 36 patients in the 240 μg group) were included in the full analysis set (FAS). From FAS, four patients were excluded whose SCr values measured after Week 4 of the treatment period were less than 5 points. The remaining patients constituted the per protocol set (PPS). PPS therefore consisted of 98 patients (34 patients in the placebo group, 29 patients in the 120 μg group and 35 patients in the 240 μg group). The safety analysis set consisted of 112 patients (35 patients in the placebo group, 36 patients in the 120 μg group and 41 patients in the 240 μg group), who received the study drug for the treatment period.

Demographic and other baseline characteristics of patients in the FAS are presented in Table 2. Baseline characteristics of patients were not significantly different among the treatment groups. It is noteworthy that 69 % to 81 % of patients were taking ARB in each group.

**Table 2:** Baseline characteristics of patients in the full analysis set (FAS)

Parameter		Treatment group		
		Placebo	120 μg	240 μg
		(n = 34)	(n = 32)	(n = 36)
Sex	Male	18 (52.9 %)	17 (53.1 %)	24 (66.7 %)
	Female	16 (47.1 %)	15 (46.9 %)	12 (33.3 %)
Age	Mean ± SD (years)	59.9 ± 10.0	56.5 ± 14.7	57.8 ± 13.9
Primary disease	Primary glomerular disease	28 (82.4 %)	27 (84.4 %)	25 (69.4 %)
	Nephrosclerosis	6 (17.6 %)	5 (15.6 %)	11 (30.6 %)
1/SCr time slope during the run-in period	Mean ± SD (dL/mg over 4 weeks)	−0.01210 ± 0.00497	−0.01535 ± 0.00808	−0.01198 ± 0.00788
SCr^a^	Mean ± SD (mg/dL)	2.377 ± 0.665	2.251 ± 0.618	2.564 ± 0.705
Urinary protein excretion^a^	Mean ± SD (mg/day)	2103.7 ± 1523.0	2037.5 ± 1763.4	1753.9 ± 1396.2
Systolic blood pressure^a^	Mean ± SD (mmHg)	122.7 ± 16.1	129.0 ± 13.3	129.4 ± 15.6
Diastolic blood pressure^a^	Mean ± SD (mmHg)	72.5 ± 11.0	73.4 ± 10.3	75.1 ± 10.6
Concomitant medication	ACEI (+)	13 (38.2 %)	9 (28.1 %)	12 (33.3 %)
ACEI/ARB positive	ARB (+)	25 (73.5 %)	26 (81.3 %)	25 (69.4 %)

^a^As of R20

### Changes in SCr

**Fig. 4:**
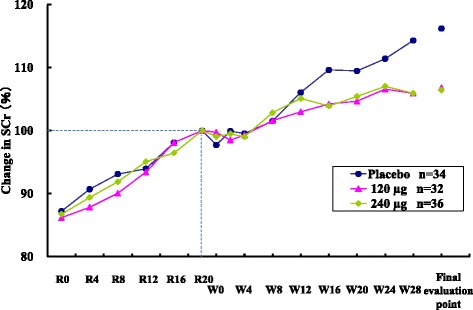
Change in SCr during study period (SCr values of each group at R20 [Week 20 of the run-in period] were set as 100 %)

Figure 4 illustrates the changes in SCr through the study periods when SCr values of each group at R20 were set at 100 %. The SCr values in all three groups increased during the run-in and treatment periods, and increases in SCr values in the treatment period tends to be suppressed in the two TRK-100STP groups.

### Efficacy

As the primary analysis set is also the FAS, the data and the results of evaluation are described based on the FAS, whilst the efficacy analysis was performed in both the FAS and PPS.

(a) Primary endpoint

*The difference in the 1/SCr versus time slope between the run-in period (R0 ~ R20) and the treatment period (W4 ~ W28)*

**Fig. 5:**
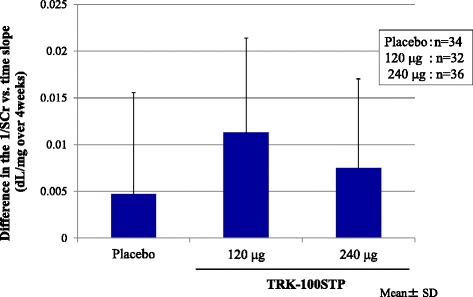
Primary endpoint: Least square mean of the change in the 1/SCr time slope

As shown in Table 3 and Fig. 5, compared with the run-in period, a slight improvement in the 1/SCr versus time slope was observed during the treatment period in all treatment groups; this tendency was clearly observed in patients of the TRK-100STP 120 μg group.

**Table 3:** Summary statistics of primary endpoint (the difference in the 1/SCr versus time slope between run-in period [R0 ~ R20] and treatment period [W4 ~ W28])

Treatment group	No. of patients	1/SCr versus time slope (dL/mg over 4 weeks)				Difference in the 1/SCr versus time slope (dL/mg over 4 weeks)	
		Run-in period (R0 ~ R20)		Treatment period (W4 ~ W28)			
		Mean	SD	Mean	SD	Mean	SD
Placebo	34	−0.0121	0.00497	−0.0074	0.00935	0.0047	0.01087
120 μg	32	−0.0154	0.00808	−0.0040	0.01345	0.0113	0.01011
240 μg	36	−0.0120	0.00788	−0.0045	0.00750	0.0075	0.00953

Table 4 shows the result of ANCOVA with the 1/SCr versus time slope during the run-in period as the covariate. No statistically significant difference was observed between the 240 μg group and the placebo group when the ANCOVA was performed with the contrast coefficients of [−1, 0, 1] for [the placebo group, the 120 μg group, and the 240 μg group] (*P* = 0.2234).

**Table 4:** Difference in the 1/SCr versus time slope between run-in period (R0 ~ R20), and Treatment period (W4 ~ W28) analyzed by the ANCOVA model: comparison of the TRK-100STP groups versus placebo

Treatment group	No. of patients	Least square mean of the change in the 1/SCr slope		Difference in the least square mean (versus placebo)^a^		Contrast test
						(*P* value)
		Point estimate	95 % CI	Point estimate	95 % CI	(−1,0,1)
Placebo	34	0.00516	[0.00187, 0.00845]	―	―	
120 μg	32	0.01026	[0.00682, 0.01369]	0.00510	[0.00031, 0.00989]	
240 μg	36	0.00798	[0.00478, 0.01118]	0.00282	[−0.00175, 0.00740]	0.2234

^a^(120 μg group or 240 μg group) – (placebo group)

1/Scr versus time slope during the run-in period as the covariate

(b) Secondary endpoints

*The difference in the 1/SCr versus time slope between the run-in period (R0 ~ R20) and the treatment period (W0 ~ W28)*

The least square mean of the difference in the 1/SCr versus time slope [95 % CI] was 0.00590 dL/mg over 4 weeks [0.00308, 0.00873] in the placebo group, 0.01065 dL/mg over 4 weeks [0.00769, 0.01360] in the TRK-100STP 120 μg group and 0.00801 dL/mg over 4 weeks [0.00525, 0.01076] in the 240 μg group. Moreover, ANCOVA was performed on the same model as the primary endpoint with the contrast coefficients of [−1, 0, 1], [−1, 1, 0], [−1, −1, 2], and [−2, 1, 1] for the placebo group, the 120 μg group, and the 240 μg group, respectively. The results of ANCOVA with contrast coefficients of [−1, 0, 1], [−1, 1, 0], [−1, −1, 2] and [−2, 1, 1] were *P* = 0.2912, *P* = 0.0244, *P* = 0.8764 and *P* = 0.0534, respectively. Thus the 120 μg group showed a significant amelioration compared with the placebo group (contrast coefficients: [−1, 1, 0]: *P* = 0.0244).

*Changes in CCr*

The difference in the change of CCr in urinalysis with 24-hour pooled urine sample (the change in the treatment period [the final evaluation point – R20]) was 1.08 ± 9.35 mL/min in the placebo group (mean ± SD), 6.16 ± 11.22 mL/min in the TRK-100STP 120 μg group and 3.94 ± 7.80 mL/min in the 240 μg group. Both TRK-100STP groups therefore showed a tendency of inhibiting the decline in CCr as compared with the placebo group (*P* = 0.0551, *P* = 0.1800: *t*-test). When CCr was calculated with the Cockcroft-Gault equation, the change in the treatment period (the final evaluation point – R20) was 2.03 ± 5.98 mL/min in the placebo group (mean ± SD), 3.93 ± 5.30 mL/min in the TRK-100STP 120 μg group and 3.77 ± 5.06 mL/min in the 240 μg group. Both TRK-100STP groups therefore showed a tendency of inhibiting the decline in CCr as compared with the placebo group (*P* = 0.1771, *P* = 0.1917: *t*-test).

*Changes in urinary protein excretion*

The change in urinary protein excretion (the final evaluation point – R20) was 121.59 ± 715.60 mg/day in the placebo group, 241.68 ± 849.23 mg/day in the TRK-100STP 120 μg group and 280.38 ± 817.97 mg/day in 240 μg group. It was shown that the observed change in the urinary protein excretion in the 120 μg and 240 μg groups (versus the placebo group) was small relative to the standard deviation, and both TRK-100STP groups showed no significant difference compared to the placebo group (*P* = 0.5457, *P* = 0.4056: *t*-test).

*Ratio of SCr*

The ratio of SCr (the final evaluation point/R20) was 1.14 ± 0.27 in the placebo group, 1.06 ± 0.20 in the TRK-100STP 120 μg group and 1.06 ± 0.13 in the 240 μg group. Although both TRK-100STP groups did not show any significant inhibition, they showed a tendency of inhibiting the increase in SCr as compared with the placebo group (*P* = 0.1379, *P* = 0.0942: *t*-test).

*Changes in serum cystatin C*

The difference in the change in serum cystatin C (the change in the treatment period [the final evaluation point – R20] – the change in the Run-in period [R20 – R0]) was 0.155 ± 0.448 mg/L in the placebo group, 0.006 ± 0.377 mg/L in the TRK-100STP 120 μg group, and −0.023 ± 0.645 mg/L in the 240 μg group. Both TRK-100STP groups therefore showed a tendency of inhibiting the increase in serum cystatin C as compared versus the placebo group (*P* = 0.1588, *P* = 0.1973: *t*-test).

(c) Post hoc analysis

*Ratio of SCr (final evaluation point/W0)*

**Fig. 6:**
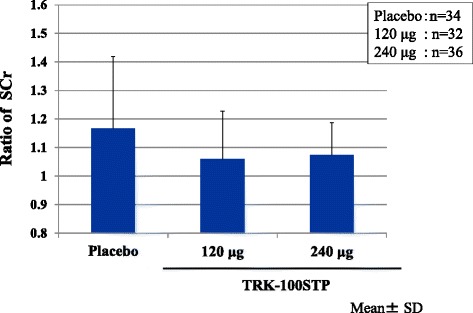
Mean ± SD of ratio of SCr (final evaluation point/W0 [week 0 of the treatment period]): As shown in Table 5, both the 120 μg and the 240 μg groups showed an inhibition of the increase in SCr ratios as compared with the placebo group (*P* = 0.0309, 0.0204, respectively), assessed by ANCOVA with the SCr (R20) as covariate

As shown in Table 5 and Fig. 6, both the 120 μg and the 240 μg groups showed a significant inhibition of the increase in SCr ratios as compared with the placebo group.

**Table 5:** Ratio of SCr [the final evaluation point^a^/W0 (week 0 of the treatment period)] analyzed by the ANCOVA model

Treatment group	Number of patients	Least square mean of the ratio of SCr		Difference in the least square mean (versus placebo)		P-value^*^
		Point estimate	SE	Point estimate	SE	
Placebo	34	1.169	0.032	–	–	–
120 μg	32	1.069	0.033	0.100	0.045	0.0309
240 μg	36	1.064	0.031	0.105	0.044	0.0204

^a^At Week 28 of the treatment period or when treatment was discontinued

*ANCOVA with baseline (SCr(R20)) as covariate

*Change in serum cystatin C in treatment period (final evaluation point – R20)*

**Fig. 7:**
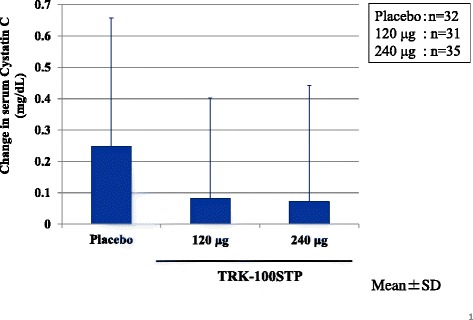
Mean ± SD of changes in serum cystatin C (final evaluation point – R20): As shown in Table 6, the 120 μg group showed a tendency and the 240 μg group showed significant inhibition of the increase in change in serum cystatin C as compared with the placebo group (*P* = 0.0928, 0.0285, respectively), assessed by ANCOVA with the SCr (R20) as covariate

As shown in Table 6 and Fig. 7, the 120 μg group showed a tendency and the 240 μg groups showed significant inhibition of the increase in change in serum cystatin C as compared with the placebo group.

**Table 6:** Changes in serum cystatin C [the final evaluation point^a^-R20 (week 20 of the Run-in period)] comparison of TRK-100STP groups versus placebo analyzed by the ANCOVA model

Treatment group	Number of patients^b^	Least square mean of the difference in serum cystatin C (mg/L)		Difference in the least square mean (versus placebo) (mg/L)		P-value
		Point estimate	SE	Point estimate	SE	
Placebo	32	0.253	0.065	―	―	―
120 μg	31	0.097	0.066	0.157	0.092	0.0928
240 μg	35	0.052	0.063	0.201	0.090	0.0285

^a^At Week 28 of the treatment period or when treatment was discontinued

^b^Different from FAS because some patients lacked value of cystatin C

### Ratio of eGFR

As shown in Table 7, the 240 μg group showed a tendency and the 120 μg groups showed significant inhibition of the decrease in eGFR ratios as compared with the placebo group.

**Table 7:** Change in eGFR [the final evaluation point^a^/W0 (week 0 of the treatment period)] analyzed by the ANCOVA model

Treatment group	Number of patients	Least square mean of the difference in serum cystatin C (mg/L)		Difference in the least square mean (versus placebo) (mg/L)		P-value
		Point estimate	SE	Point estimate	SE	
Placebo	34	0.877	0.025	―	―	―
120 μg	32	0.952	0.026	0.075	0.036	0.0365
240 μg	36	0.942	0.024	0.065	0.035	0.0628

^a^At Week 28 of the treatment period or when treatment was discontinued

### Safety and tolerability

The safety analysis was conducted based on any AEs reported during the study period. The incidence of AEs are summarized in Table 8. Two death were observed in this study; they occurred during the run-in period and so any causal relationships with the study drug was excluded.

**Table 8:** Summary of the incidence of adverse events

Parameter	120 μg	240 μg	Placebo
Number of patients evaluated	36	41	35
Number of patients who experienced adverse events	27 (75.0 %)	36 (87.8 %)	28 (80.0 %)
Number of patients who experienced serious adverse events	3 (8.3 %)	4 (9.8 %)	1 (2.9 %)
Number of patients who discontinued the study treatment due to adverse events	4 (11.1 %)	7 (17.1 %)	0 (0.0 %)
Number of patients who interrupted the study treatment due to adverse events	0 (0.0 %)	2 (4.9 %)	0 (0.0 %)

(): Incidence rate

The incidence of ADRs are summarized in Table 9. All of these ADRs cases were mild to moderate in severity, and recovered without treatment. Two serious adverse drug reactions (SADR) of cardiac failure and acute myocardial infarction were observed in the 120 μg group after randomization and both patients recovered.

**Table 9:** Summary of adverse drug reaction incidence

Parameter	120 μg	240 μg	Placebo
Number of patients evaluated	36	41	35
Number of patients who experienced ADR	7 (19.4 %)	13 (31.7 %)	5 (14.3 %)
Number of patients who experienced serious ADR	2 (5.6 %)	0 (0.0 %)	0 (0.0 %)
Number of patients who discontinued the study treatment due to ADR	3 (8.3 %)	5 (12.2 %)	0 (0.0 %)
Number of patients who interrupted the study treatment due to ADR	0 (0.0 %)	1 (2.4 %)	0 (0.0 %)

(): Incidence rate

The AEs and ADRs that occurred at an incidence of 5 % or more are summarized in Table 10. The severity of headache in ADR was as follows: moderate headache was reported in 1 patient in the 240 μg group; mild headache was reported in 1 patient in the 120 μg group, and 5 patients in the 240 μg group. All of these patients recovered without treatment. For clinical laboratory tests, vital signs, body weight and 12-lead ECG, no specific concerns were observed. TRK-100STP did not have significant effect on blood pressure at any dose, as detailed in Table 11.

**Table 10:** Adverse events and adverse drug reactions with an incidence of 5 % or more

Adverse Events					
120 μg		240 μg		Placebo	
Headache	5 (13.9 %)	Nasopharyngitis	11 (26.8 %)	Nasopharyngitis	8 (22.9 %)
Nasopharyngitis	4 (11.1 %)	Headache	7 (17.1 %)	Pruritus	3 (8.6 %)
Back pain	3 (8.3 %)	Diarrhea	5 (12.2 %)	Fever	3 (8.6 %)
Hyperkalemia	2 (5.6 %)	Malaise	3 (7.3 %)	Hyperkalemia	3 (8.6 %)
Dizziness	2 (5.6 %)			Influenza	2 (5.7 %)
Hypertension	2 (5.6 %)			Hyperkalemia	2 (5.7 %)
Upper respiratory tract inflammation	2 (5.6 %)			Muscle Spasm	2 (5.7 %)
Diarrhea	2 (5.6 %)			Genital Bleeding	2 (5.7 %)
Vomiting	2 (5.6 %)				
Arthralgia	2 (5.6 %)				
Adverse drug reactions					
120 μg		240 μg		Placebo	
Hypertension	2 (5.6 %)	Headache	6 (14.6 %)	Genital Bleeding	2 (5.7 %)
		Malaise	3 (7.3 %)		

(): Incidence rate

**Table 11:** Effect of TRK-100STP 120 μg and 240 μg on blood pressure

Treatment group		Systolic blood pressure		Diastolic blood pressure	
		R20	Final evaluation point	R20	Final evaluation point
Placebo	No. of patients	35	35	35	35
	Mean ± SD	123.8 ± 17.0	127.5 ± 20.3	72.7 ± 10.9	75.3 ± 9.9
120 μg	No. of patients	36	35	36	35
	Mean ± SD	128.3 ± 13.0	127.8 ± 13.5	73.6 ± 10.7	75.4 ± 10.5
240 μg	No. of patients	41	40	41	40
	Mean ± SD	128.3 ± 16.4	129.4 ± 13.7	74.4 ± 10.6	75.1 ± 8.9

(Unit of blood pressure: mmHg)

## Discussion

This is the first randomized, double-blind, placebo controlled comparative trial of TRK-100STP to investigate the possible therapeutic dose of TRK-100STP in CKD patients with a primary disease of glomerular disease or nephrosclerosis.

In the FAS, statistical superiority of the 240 μg group over the placebo could not be confirmed by the primary endpoint, and so the putative recommended dose could not be clearly determined. One of the reasons why statistically significant superiority could not be confirmed was the trial lost statistical power due to the fact that more patients than had been expected were withdrawn from the study during the run-in period. As we needed to consider the feasibility of study completion, we could not secure the sufficient number of patients for analysis.

This is considered to be because the pace at which the decline in renal function became slower during the run-in period. As shown in Table 3, 1/SCr versus time slope in the treatment period was ameliorated even in the placebo group. In this trial, the CKD patients who progressed and whose 1/SCr versus time slopes were ≤ −0.005 dL/mg over 4 weeks were selected at the start of the treatment period. As a result, the number of patients to be enrolled was reduced to about 1/3 of patients who had been screened. It is thought that such selection of patients also led to the regression toward the mean in all groups. This amelioration in the 1/SCr versus time slope in the placebo group may have made it difficult to detect the difference between the placebo group and the 240 μg group in terms of the primary endpoint. However, our trial showed, for the first time, that TRK-100STP might prevent the decline of renal function. This conclusion was supported by the following facts: both active study drug treatment groups exhibited better efficacy than the placebo group, and the efficacy was particularly high in the 120 μg group on the primary endpoint. In the secondary endpoint analysis, both TRK-100STP groups showed improvements on each of the renal filtration function parameters evaluated (1/SCr versus time slope, change in the CCr, ratio of SCr, and change in serum cystatin C). Among them, a significant improvement was observed in the slope for 1/SCr versus time when contrast coefficients [−1, 1, 0] were used, suggesting a valid hypothesis that the improvement in 120 μg group was remarkable. In addition, in the post-hoc analysis, a significant improvement in the ratio of SCr was achieved in the 120 μg group as well as in the 240 μg group. The difference in cystatin C levels in the treatment period also improved in the 240 μg group. Regarding the eGFR ratio, a significant improvement was observed only in the 120 μg group.

Significant effects on blood pressure or urinary protein levels were not observed in this study, and thus the mechanisms of action of BPS are thought to be different from those of existing agents such as ACEIs and ARBs. Since the majority of patients in this study was taking ACEIs and/or ARBs, TRK-100STP may be a useful for treatment of CKD patients in combination with ACEIs or ARBs. TRK-100STP was considered to be well tolerated. Most of the AEs observed in this study were similar types of the events caused by prostacyclin and its analogue.

Several important limitations to this study are apparent: as the treatment period was comparatively short (28 weeks) for evaluating the decrease in the renal function, the enrollment to the trial was restricted to those patients whose renal function had progressively declined. In addition, no dialysis or doubling of SCr was observed during the study period so that its efficacy on the renal composite endpoint including dialysis was unclear. Further evaluation in a longer study period will therefore be necessary in future.

Based on the findings of this trial, we have further been conducting a phase IIb/III trial, CASSIOPEIR (**C**RF **As**ian **S**tudy w**i**th **O**ral **P**GI_2_ derivative for **E**valuating **I**mprovement of **R**enal function) [42]. This new trial will enroll patients who have more severe disease than those enrolled in this trial in order to ensure that a sufficient number of clinical events can be observed within a designated time period, using renal composite endpoints to evaluate treatment outcome. This trial will also be dose-finding as it utilizes the same two doses as used in this study. The trial is being conducted in seven Asian countries, including Japan.

## Conclusions

Although this randomized, placebo-controlled, double-blind, comparative trial failed to meet the primary endpoint to determine the recommended dose for the treatment of patients with CKD with primary glomerular disease or nephrosclerosis, it shows for the first time, that TRK-100STP might potentially prevent the decline in renal function of such patients, independent of blood pressure or urinary protein levels. These findings have helped in the design and conduct of the ongoing phase IIb/III trial, CASSIOPEIR.

## Additional file

Additional file 1:
**CONSORT 2010 Flow Diagram.** (DOC 37 kb)

## Abbreviations

ACEIs: Angiotensin-converting enzyme inhibitors
ADR: Adverse drug reaction
AE: Adverse event
ANCOVA: Analysis of covariance
ARB: Angiotensin-II receptor blocker
BPS: Beraprost sodium
CASSIOPEIR: **C**RF **As**ian **S**tudy w**i**th **O**ral **P**GI_2_ derivative for **E**valuating **I**mprovement of **R**enal function
CCr: Creatinine clearance
CKD: Chronic kidney disease
CRF: Chronic renal failure
ECG: Electrocardiogram
ESRD: End-stage renal disease
FAS: Full analysis set
NSAIDs: Nonsteroidal anti-inflammatory drugs
PGI_2_: Prostaglandin I_2_ (also called prostacyclin)
PPS: Per protocol set
RAS: Renin-angiotensin system
SADR: Serious adverse drug reactions
SCr: Serum creatinine
