# Supplementary material for: Orally active prostacyclin analogue beraprost sodium in patients with chronic kidney disease: a randomized, double-blind, placebo-controlled, phase II dose finding trial
Source: BMC Nephrol. 2015 Oct 16;16:165. doi: 10.1186/s12882-015-0130-5 (PMC4608181; doi:10.1186/s12882-015-0130-5)
Supplement: Additional file 1: — CONSORT 2010 Flow Diagram. (DOC 37 kb) [file 12882_2015_130_MOESM1_ESM.doc]

**CONSORT 2010 Flow Diagram**

**Allocation**

**Analysis**

**Follow-Up**

**Enrollment**

Lost to follow-up (give reasons) (n=0)

Discontinued intervention (give reasons) (n=7)

• Adverse event: n = 6

• Unsatisfactory response: n = 1

Assessed for eligibility (n=431 )

Excluded (n=319 )

♦  Not meeting inclusion criteria (n=252 )

♦  Declined to participate (n=22 )

♦  Other reasons (n=45 )

Analysed (n=36)
♦ Excluded from analysis (give reasons) (n=0)

Lost to follow-up (give reasons) (n=0)

Discontinued intervention (give reasons) (n=8)

•Adverse event: n = 3

•Protocol deviations: n = 3

•Target disease exacerbation (side effect): n = 1

•Not meeting inclusion/exclusion criteria: n = 1

Allocated to intervention (n= 36)

♦ Received allocated intervention (n=36)

♦ Did not receive allocated intervention (give reasons) (n=0 )

Lost to follow-up (give reasons) (n=0)

Discontinued intervention (give reasons) (n=3)

• Protocol deviation: n =1

• Unsatisfactory response: n =1

• Target disease exacerbation: n =1

Allocated to intervention (n= 35)

♦ Received allocated intervention

(n= 35 )

♦ Did not receive allocated intervention (give reasons) (n=0 )

Analysed (n=35)
♦ Excluded from analysis (give reasons) (n=0)

Randomized (n=112 )

Allocated to intervention (n= 41)

♦ Received allocated intervention (n=41)

♦ Did not receive allocated intervention (give reasons) (n=0 )

Analysed (n=41)
♦ Excluded from analysis (give reasons) (n=0)
